# Association of Circadian Rhythm Biomarkers and Ambulatory Blood Pressure Monitoring With Prognosis and Outcome in People With Acute Ischemic Stroke: A Case-Control Study

**DOI:** 10.7759/cureus.89222

**Published:** 2025-08-01

**Authors:** Virendra Atam, Nikhil K Gupta, Manohar K Jha, Isha Atam, Jay Tewari, Vanshika Singh, Ambuj Yadav, Gyanendra K Sonkar, Deeksha Chahar, Pankhuri Singh

**Affiliations:** 1 Internal Medicine, King George's Medical University, Lucknow, IND; 2 Physiology, King George's Medical University, Lucknow, IND; 3 Biochemistry, King George's Medical University, Lucknow, IND

**Keywords:** acute ischemic stroke, ambulatory blood pressure monitoring, cortisol, melatonin, outcomes, prognosis

## Abstract

Introduction

This study examines the relationship between circadian biomarkers-specifically salivary cortisol and urinary melatonin-and ambulatory blood pressure monitoring (ABPM) patterns in patients with acute ischemic stroke (AIS). The goal was to determine whether these biomarkers could serve as prognostic indicators for stroke severity and recovery outcomes using the National Institutes of Health (NIH) Stroke Scale (NIHSS) and the modified Rankin scale (MRS) at discharge.

Materials and methods

This case-control study enrolled 75 AIS patients and 75 age- and sex-matched controls at a tertiary care center in northern India. Salivary cortisol and urinary melatonin were measured using enzyme-linked immunosorbent assay (ELISA) at a pre-specified time of the day. ABPM was performed over 24 hours. Stroke severity was assessed using the NIHSS, and functional outcomes were evaluated using the MRS. Statistical analyses included t-tests, Chi-squared tests, ANOVA, and Pearson correlation.

Results

In a comparative analysis involving 75 stroke patients and 75 controls, stroke patients demonstrated significantly lower urinary melatonin levels compared to controls, indicating a disruption in circadian rhythm. Although salivary cortisol levels were higher in stroke cases, the difference was not significant. ABPM data revealed a reduction in normal “dipper” patterns among stroke patients, with an increase in non-dipper and reverse-dipper profiles. Notably, lower melatonin levels were strongly associated with higher stroke severity and increased disability scores, while elevated cortisol levels correlated with greater stroke severity.

Conclusions

Alterations in circadian biomarkers, particularly reduced melatonin and modestly elevated cortisol, are linked to increased stroke severity and poorer functional outcomes. Additionally, abnormal ABPM patterns may reflect underlying autonomic dysregulation associated with stroke prognosis. These findings suggest that circadian biomarkers that were elaborated in this study could provide valuable insights for predicting stroke recovery and may offer new targets for therapeutic intervention.

## Introduction

Circadian rhythms, regulated by the suprachiasmatic nucleus (SCN) of the hypothalamus, are essential biological processes that control various physiological functions. These include the secretion of hormones such as melatonin and cortisol released from the pineal and adrenal glands, respectively. Melatonin plays a crucial role in neuroprotection due to its antioxidative, anti-inflammatory, and antiapoptotic properties. By scavenging free radicals and reducing oxidative stress, melatonin helps mitigate neuronal damage caused by ischemic stroke [1-3]. Additionally, it modulates mitochondrial function, thereby preventing neuronal apoptosis and preserving cellular integrity [4]. Beyond its neuroprotective effects, melatonin directly influences vascular health and blood pressure (BP) regulation. Low melatonin levels have been linked to nocturnal hypertension, a well-recognized risk factor for stroke severity and poor functional outcomes [5,6]. Cortisol, a glucocorticoid hormone, plays a crucial role in mediating the body’s stress response, and its dysregulation significantly impacts stroke pathophysiology. Elevated cortisol levels are associated with endothelial dysfunction, increased BP, and a pro-inflammatory state, all of which exacerbate stroke outcomes [7]. Beyond its cardiovascular effects, cortisol profoundly influences neurovascular function. It disrupts cerebral blood flow regulation and compromises the integrity of the blood-brain barrier, heightening the risk of secondary damage following a stroke, as was found in a study using diagnostic models with genes [8]. “Acute” typically refers to the period within the first seven days post-stroke, while “subacute” generally encompasses the period from seven days up to three months post-stroke. Furthermore, chronic dysregulation of the hypothalamic-pituitary-adrenal axis has been linked to post-stroke cognitive impairment, depression, and delayed functional recovery. Clinical studies indicate that people with persistently high salivary cortisol levels during the acute and subacute phases of stroke tend to suffer from more severe neurological deficits and poorer long-term recovery [7]. BP fluctuations follow a circadian rhythm, typically marked by a nocturnal dip. Disruptions in this pattern, such as non-dipping or reverse dipping BP, have significant implications for stroke risk and recovery [9]. Persistent hypertension or excessive BP variability is linked to larger infarcts and poorer functional outcomes, while post-stroke BP dysregulation is associated with an increased risk of hemorrhagic transformation and secondary complications. Ambulatory BP (ABP) monitoring (ABPM) in stroke patients offers valuable prognostic insights, and targeted antihypertensive therapy based on ABP patterns may enhance long-term outcomes [10].

Acute ischemic stroke (AIS) is a leading cause of morbidity and mortality worldwide, and there is growing evidence that circadian markers such as urinary melatonin, salivary cortisol, and ABPM could offer valuable prognostic insights. Their integration into stroke management could enhance risk stratification, inform personalized therapeutic strategies, and improve long-term outcomes; such treatment and management modalities should be further investigated [11]. Despite the increasing evidence highlighting the significance of circadian rhythms in stroke, several research gaps persist. This study examines the relationship between these circadian markers and stroke outcomes, analyzing their potential role in predicting prognosis and guiding clinical management.

## Materials and methods

This was a case-control study conducted over a period of six months (December 2024 to May 2025) in the Department of Internal Medicine at King George's Medical University (KGMU) in Lucknow, Uttar Pradesh, India. The study was approved by the Institutional Ethics Committee (Ref. code: XXVII-PGTSC-IIA/P78).

People admitted to the Department of Internal Medicine at KGMU who met the inclusion criteria and provided written informed consent were enrolled in the study as cases. The inclusion criteria were age over 12 years, acute stroke meeting the World Health Organization (WHO) definition of stroke, and admission within six hours of the stroke episode. The exclusion criteria were hemorrhagic stroke, clinical and radiological findings inconsistent with stroke, head injury, intracranial space-occupying lesion, and chronic kidney disease. Controls were people without stroke who attended the Internal Medicine outpatient clinic and consented to participate in the study.

All participants underwent the following procedures in the hospital after providing informed consent: detailed history-taking, clinical examination, and stroke severity assessment using the National Institutes of Health Stroke Scale (NIHSS) score; computed tomography scan of the brain; laboratory investigations: complete blood count, random blood glucose, serum electrolytes, kidney and liver function tests, echocardiogram, lipid profile, prothrombin time, international normalized ratio, routine urine analysis, echocardiography (if cardioembolic stroke was suspected), and chest x-ray; salivary cortisol measurement using a competitive inhibition enzyme-linked immunosorbent assay (ELISA) (CEA462Ge 96 tests) between 6:00 AM and 9:00 AM; urinary melatonin measurement using a commercial ELISA kit (Human Melatonin Sulfate ELISA Kit) between 8:00 PM and 10:00 PM; and ABPM for 24 hours using a validated, automated device (Microlife WatchBP O3 (Widnau, Switzerland)) to evaluate the circadian BP profile. BP readings were recorded at 30-minute intervals during the daytime (6:00 AM to 10:00 PM) and 60-minute intervals during the nighttime (10:00 PM to 6:00 AM). The patients were instructed to maintain their usual activity and sleep routines.

Data were entered into Microsoft Excel (Microsoft Corp., Redmond, WA, US) and analyzed using SPSS version 29.0.2.0 (IBM Corp., Armonk, NY, US). Continuous variables were presented as mean (standard deviation), and dichotomous variables were presented as number (frequency). The Chi-squared test was used to analyze dichotomous variables, and Student's t-test was used to compare means between groups. A p-value of less than 0.05 was considered statistically significant.

## Results

A total of 75 cases and 75 controls were enrolled in this study. The mean age of the stroke cases was significantly higher compared to the controls, with the difference being statistically significant (t = 3.502, p = 0.0007), revalidating the obvious that age is a risk factor for stroke. Males constituted 52.0% of the case group and 72.0% of the control group. The difference in gender distribution between the groups was also statistically significant (χ² = 6.367, p = 0.0116), indicating a disproportionate representation of males in the control group.

Hypertension was significantly more prevalent among cases (n = 48, 64.0%) compared to controls (n = 35, 46.7%), and this difference was highly statistically significant (χ² = 37.14, p < 0.0001), reinforcing its role as a major risk factor for AIS. Type 2 diabetes mellitus (T2DM) was also more common among cases (n = 32, 42.7%) than controls (n = 18, 24.0%). Dyslipidemia was slightly more common in controls (n = 36, 48.0%) than cases (n = 30, 40.0%), while smoking prevalence was similar in both groups (n = 21, 28.0% in cases vs. n = 20, 26.7% in controls). Notably, alcohol consumption was substantially higher in cases (n = 4, 5.3%) than in controls (n = 1, 1.33%). These have been shown in Table 1.

**Table 1:** Demographic characteristics and comorbidities in the cases and controls SD: standard deviation; df: degree of freedom; Cohen’s d and Cramér’s V: effect sizes

	Cases (mean ± SD/N = 75)	Controls (mean ± SD/N = 75)	p-value
Age (years)	62.67 ± 12.43	54.23 ± 13.93	t = 3.502, p = 0.0007, Cohen’s d = 0.64, df = 148
Male	39 (52.0%)	54 (72.0%)	χ^2^ = 6.367, p = 0.0116, Cramér’s V = 0.206, df = 1
Female	36 (48.0%)	21 (28.0%)	
Comorbidities			
Hypertension	48 (64.0%)	35 (46.7%)	χ^2^ = 37.14, p < 0.0001, Cramér’s V = 0.498, df = 4
Type 2 diabetes mellitus	32 (42.7%)	18 (24.0%)	
Dyslipidemia	30 (40.0%)	36 (48.0%)	
Smoking	21 (28.0%)	20 (26.7%)	
Alcohol consumption	4 (5.3%)	1 (1.33%)	

**Figure 1:**
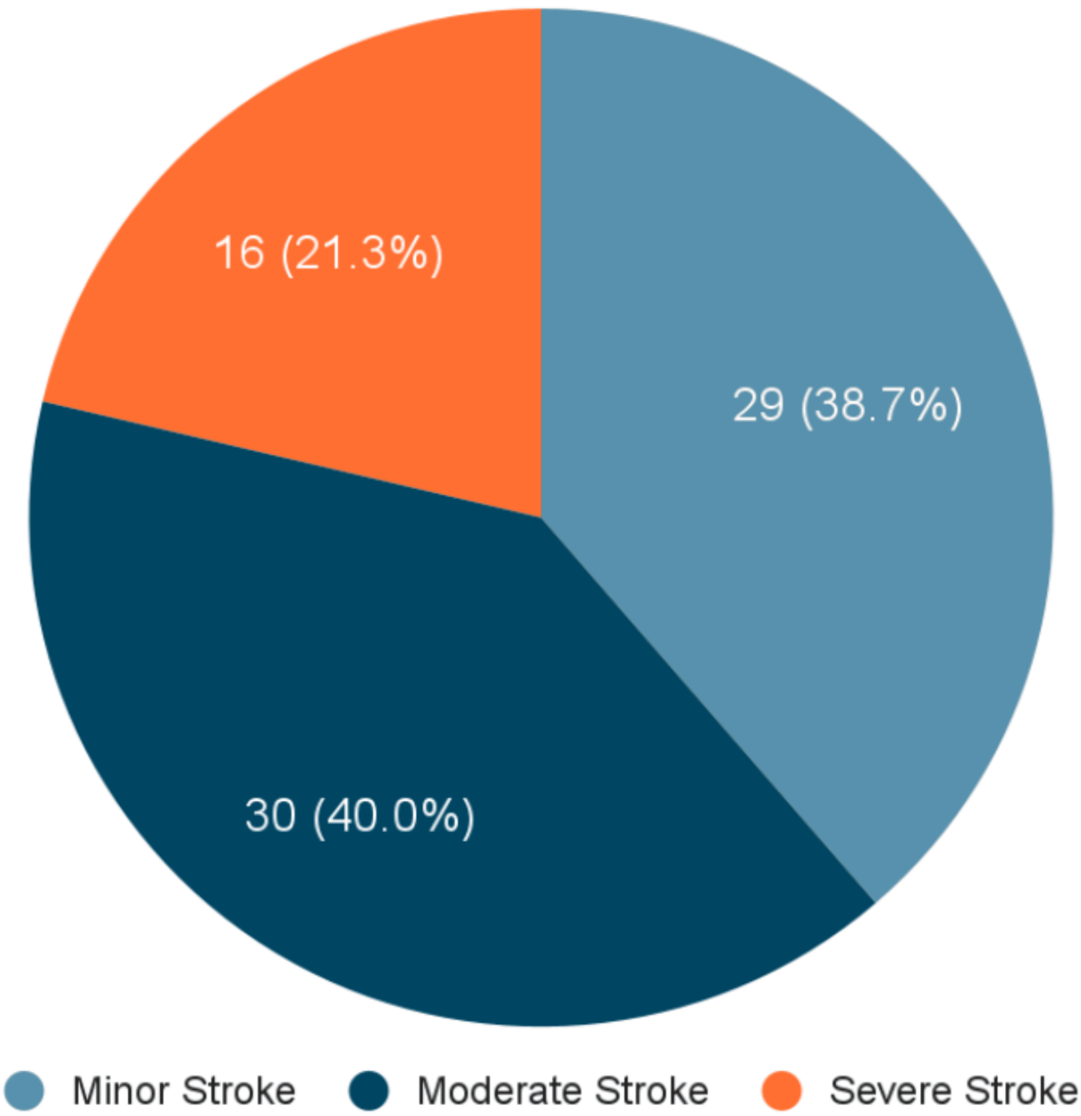
Distribution of stroke cases as per the NIHSS score NIHSS: National Institutes of Health Stroke Scale

The majority of people had mild to moderate strokes, with 29 people (38.7%) classified as having minor stroke and 30 people (40.0%) as having moderate stroke. Severe strokes were less common, accounting for 16 patients (21.3%). The mean NIHSS score was 11.07 ± 8.05, indicating that the average patient experienced a moderate neurological deficit at presentation. This has been shown in Figure 1.

**Figure 2:**
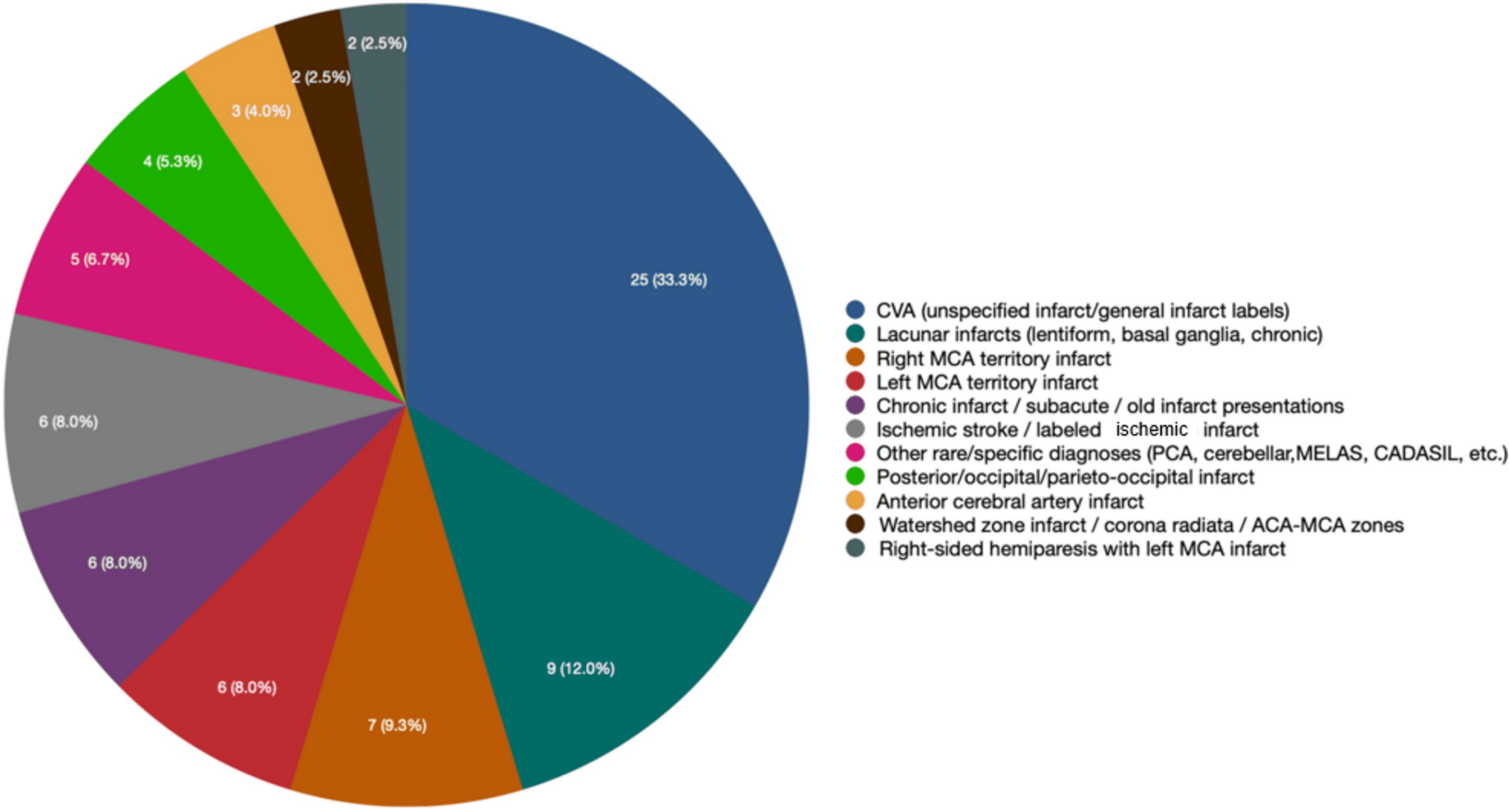
Diagnosis distribution among stroke cases CVA: cardiovascular accident; MCA: middle cerebral artery; PCA: posterior cerebral artery; MELAS: mitochondrial encephalomyopathy, lactic acidosis, and stroke-like episodes; CADASIL: cerebral autosomal dominant arteriopathy with subcortical infarcts and leukoencephalopathy; ACA-MCA: anterior cerebral artery-middle cerebral artery

Figure 2 shows the territorial distribution of strokes in the cases. The most common diagnosis was broadly labeled as cardiovascular accident (CVA) or unspecified infarct, accounting for 33.3% of cases (n = 25), reflecting non-specific clinical or radiological documentation. Lacunar infarcts involving the lentiform nucleus or basal ganglia were next most frequent (n = 9, 12%), followed by right middle cerebral artery (MCA) territory infarcts (n = 7, 9.3%). The left MCA territory infarct, chronic infarct/subacute/old infarct presentations, and ischemic stroke/labeled ischemic infarct accounted for 8% (n = 6) of the cases each. Other rare/specific diagnoses (n = 5, 6.7%), posterior, occipital, or parieto-occipital infarcts (n = 4, 5.3%), anterior cerebral artery (ACA) infarct (n = 3, 4%), watershed zone infarct/corona radiata/ACA-MCA zones (n = 2, 2.5%), and right-sided hemiparesis with left MCA infarct (n = 2, 2.5%) were among the less frequently observed diagnoses in the cases.

A statistically significant improvement in modified Rankin scale (MRS) scores was observed over the hospital course (χ² = 10.39, p = 0.0010), as shown in Table 2. We measured the MRS at admission and on discharge. At admission, the majority of patients had severe disability (MRS 5), accounting for 52.0% (n = 39) of cases, which decreased notably to 22.7% (n = 17) at discharge. Conversely, those with milder disability (MRS 1 to 3) increased substantially by discharge-MRS 1 rose from 2.7% (n = 2) to 10.7% (n = 8), MRS 2 from 8.0% (n = 6) to 13.3% (n = 10), and MRS 3 from 10.7% (n = 8) to 26.7% (n = 20). Patients in the MRS 4 category remained constant at 26.7% (n = 20), suggesting some cases with persistent moderate-to-severe disability.

**Table 2:** Modified Rankin scale (MRS)–admission vs. discharge *: statistically significant result; df: degree of freedom; Cramér’s V: effect size

MRS score	Admission (n, %)	Discharge (n, %)	p-value
1	2 (2.7%)	8 (10.7%)	χ^2^ = 10.39, p = 0.0010*, Cramér’s V = 0.372, df = 4
2	6 (8.0%)	10 (13.3%)	
3	8 (10.7%)	20 (26.7%)	
4	20 (26.7%)	20 (26.7%)	
5	39 (52.0%)	17 (22.7%)	

The mean salivary cortisol level was substantially higher in cases compared to controls; however, this difference was not statistically significant (t = 0.5317, p = 0.5959), likely due to the large variability among cases, as reflected by the wide standard deviation. In contrast, the mean urinary melatonin level was significantly lower in stroke cases (7.18 ± 1.91 ng/mL) than in controls (14.26 ± 1.98 ng/mL), and this difference was very highly significant (t = 19.93, p < 0.0001).

Among controls, 96.0% (n = 72) demonstrated a normal dipper pattern, indicating the expected nocturnal decline in BP. In contrast, only 52.0% (n = 39) of stroke cases exhibited this dipper pattern. Instead, a substantial 44.0% (n = 33) of stroke patients showed a non-dipper pattern, where BP fails to fall at night-a known risk factor for cerebrovascular events. Additionally, 4.0% (n = 3) of cases displayed a reverse dipping pattern, where nighttime BP paradoxically increases, further heightening stroke risk. No controls showed reverse dipping. This has been shown in Table 3.

**Table 3:** Circadian rhythm markers and ambulatory blood pressure monitoring patterns in the cases SD: standard deviation; df: degree of freedom; Cohen’s d and Cramér’s V: effect sizes

Parameter	Cases (mean ± SD)	Controls (mean ± SD)	p-value
Salivary cortisol (ng/mL)	150.63 ± 93.52	20.21 ± 6.64	t = 0.5317, p = 0.5959, df = 148, Cohen's d = 1.97
Urinary melatonin (ng/mL)	7.18 ± 1.91	14.26 ± 1.98	t = 19.93, p < 0.0001, df = 148, Cohen's d = −3.64
Dipper	39 (52.0%)	72 (96.0%)	χ² = 37.81, p < 0.0001, df = 2, Cramér’s V = 0.502
Non-dipper	33 (44.0%)	3 (4.0%)	
Reverse dipping	3 (4.0%)	0 (0.0%)	

Among the three ABPM categories-dipper, non-dipper, and reverse dipper-the distribution of MRS scores shows no statistically significant association (χ² = 3.443, p = 0.9036). While a slightly higher proportion of dippers had favorable outcomes (MRS 1 or 2: n = 12, 30.7%) compared to non-dippers (n = 6, 16.2%), this difference was statistically insignificant. Notably, all reverse dippers (n = 3) had poor outcomes (MRS 3-5), but the very small sample size limits generalizability.

We speculate that salivary cortisol levels tend to be higher among patients with better outcomes (MRS 1: 211.68 ± 116.53 ng/mL) and fluctuate inconsistently across worse scores, indicating an acute stress response or possible compensatory hormonal surge in early recovery. The statistical analysis shows a significant variation across MRS categories (F = 8.30, p < 0.0001), confirming that cortisol levels are meaningfully associated with functional status. In contrast, urinary melatonin levels demonstrate an inverse relationship with MRS scores-being highest in patients with minimal disability (MRS 2: 9.67 ± 1.8 ng/mL) and lowest in those with greater disability (MRS 1: 5.35 ± 0.46 ng/mL). The variation is very highly statistically significant (F = 42.03, p < 0.0001), suggesting that melatonin levels are strongly associated with functional outcomes, likely reflecting preserved circadian rhythm integrity in patients with better recovery. This has been shown in Table 4.

**Table 4:** Association of ABPM patterns with MRS score at discharge and comparison of salivary cortisol and urinary melatonin levels across MRS scores at discharge MRS: modified Rankin scale; *: statistically significant result; Eta squared η^2^ and Cramér’s V: effect sizes; ABPM: ambulatory blood pressure monitoring

MRS score at discharge	Dipper (n, %)	Non-dipper (n, %)	Reverse dipping (n, %)	p-value	Salivary cortisol (10-28 ng/mL)	p-value	Urinary melatonin	p-value
1	5 (12.8%)	3 (8.1%)	0 (0.0%)	χ² = 3.443, p = 0.9036, df = 8, Cramér’s V = 0.151	211.68 ± 116.53	F = 8.30; p < 0.0001*, df = 4.70; Eta squared η^2^ = 0.322	5.35 ± 0.46	F = 42.03; p < 0.0001*, df = 4.70; Eta squared η^2^ = 0.706
2	7 (17.9%)	3 (8.1%)	0 (0.0%)		62.3 ± 78.35		9.67 ± 1.8	
3	10 (25.6%)	9 (24.3%)	1 (33.3%)		136.5 ± 63.73		7.38 ± 1.68	
4	8 (20.5%)	11 (29.7%)	1 (33.3%)		169.3 ± 95.63		6.52 ± 1.16	
5	9 (23.1%)	7 (18.9%)	1 (33.3%)		168.53 ± 72.27		6.98 ± 1.76	
Total	39	33	3					

The distribution across dipper, non-dipper, and reverse dipping groups did not show a statistically significant association (χ² = 2.411, p = 0.6607), indicating that ABPM pattern was not a strong predictor of stroke severity at presentation. Among dipper patients, 43.6% (n = 17) had minor strokes, which was higher than those in non-dippers (32.4%, n = 12) and absent in reverse dippers. Conversely, reverse dippers had a disproportionately higher rate of moderate (66.7%, n = 2) and severe (33.3%, n = 1) strokes, though based on a very small sample (n =3), limiting conclusive interpretation. The findings have been listed in Table 5.

**Table 5:** Association of ABPM patterns with NIHSS severity df: degree of freedom; Cramér’s V: effect sizes; NIHSS: National Institutes of Health Stroke Scale; ABPM: ambulatory blood pressure monitoring

NIHSS stroke severity	Dipper (n, %)	Non-dipper (n, %)	Reverse dipping (n, %)	p-value
Minor stroke	17 (43.6%)	12 (32.4%)	0 (0.0%)	χ² = 2.411, p = 0.6607, df = 4, Cramér’s V = 0.127
Moderate stroke	14 (35.9%)	14 (37.8%)	2 (66.7%)	
Severe stroke	8 (20.5%)	7 (18.9%)	1 (33.3%)	
Total (n)	39	33	3	

A clear trend is observed in salivary cortisol levels, which increase with stroke severity (according to the NIHSS score)-from 113.70 ± 95.15 ng/mL in minor strokes to 176.19 ± 59.17 ng/mL in severe strokes. This pattern is statistically significant (F = 3.44, p = 0.0366), indicating that higher cortisol levels may be associated with worse neurological outcomes, possibly due to heightened stress and neuroendocrine dysregulation in more severe strokes. Conversely, urinary melatonin levels show a significant decline as stroke severity increases-highest in minor strokes (8.08 ± 2.25 ng/mL) and lowest in severe strokes (6.18 ± 1.11 ng/mL). This association is very highly significant (F = 8.35, p < 0.0001). These findings have been listed in Table 6.

**Table 6:** Comparison of salivary cortisol and urinary melatonin levels according to stroke severity based on NIHSS scores at discharge NIHSS: National Institutes of Health Stroke Scale; *: statistically significant result; Eta squared: effect sizes

NIHSS score (at discharge)	Salivary cortisol (10-28 ng/mL)	Urinary melatonin
Minor stroke	113.70 ± 95.15	8.08 ± 2.25
Moderate stroke	172.70 ± 93.81	6.77 ± 1.40
Severe stroke	176.19 ± 59.17	6.18 ± 1.11
p-value	F = 3.44; p = 0.0366*, df = 2.72; Eta squared = 0.0872	F = 8.35; p < 0.0001*, df = 2.72; Eta squared = 0.188

**Figure 3:**
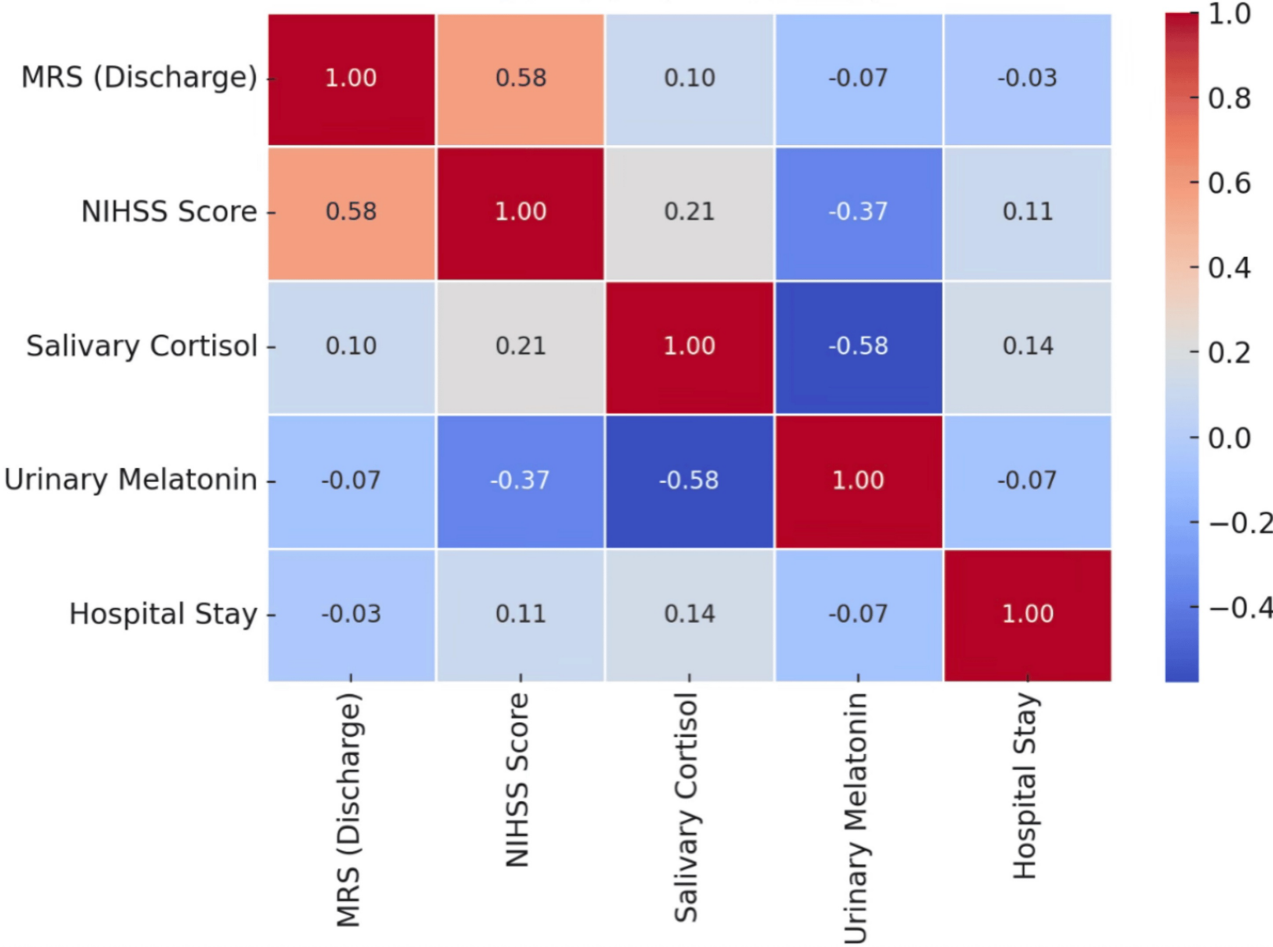
Pearson correlation coefficients examining the relationships between functional and neurological outcomes (MRS at discharge, NIHSS score), circadian biomarkers (salivary cortisol and urinary melatonin), and duration of hospitalization in stroke patients NIHSS: National Institutes of Health Stroke Scale; MRS: modified Rankin scale

In Figure 3, a strong positive correlation was found between NIHSS and MRS at discharge (r = 0.583, p = 0.0001), indicating that greater stroke severity is linked to higher disability. Salivary cortisol showed weak, non-significant correlations with both MRS (r = 0.101, p = 0.389) and NIHSS (r = 0.215, p = 0.064), suggesting limited association. Urinary melatonin had a moderate-to-strong negative correlation with NIHSS (r = -0.373, p = 0.001) and cortisol (r = -0.578, p = 0.000), indicating that lower melatonin is linked to greater stroke severity and stress. Hospitalization duration showed no significant correlation with any variables, implying it is influenced by factors such as logistics, comorbidities, or institutional protocols.

## Discussion

This case-control study was conducted to evaluate the association between markers of circadian rhythm-namely, salivary cortisol and urinary melatonin-and BP variability patterns obtained through ABPM with the clinical outcomes of patients suffering from AIS. In the case group, males comprised 52.0%; conversely, in the control group, males were substantially more predominant, representing 72.0% of participants. This uneven gender distribution suggests that while stroke may affect both sexes, the current sample had a higher female representation among cases. It may also point toward underdiagnosis or differential health-seeking behavior between genders in the control population.

Chronic elevation of BP contributes to endothelial injury, promotes atherosclerosis, and predisposes to thromboembolic events, making it an important risk factor for AIS. Diabetes accelerates atherosclerosis, induces a prothrombotic state, and leads to microvascular damage, which together heightens the risk of both ischemic events and poor post-stroke recovery [12]. The additive risk posed by coexisting hypertension and diabetes in stroke patients is especially critical, as both conditions interact synergistically to exacerbate vascular injury. Alcohol intake and smoking did not differ significantly between groups. Interestingly, dyslipidemia showed a reversed trend, being slightly more frequent among controls (48.0%) than in cases (40.0%). Alternatively, an acute phase response during stroke may result in transient suppression of lipid levels, thereby underestimating true dyslipidemic status [13].

The mean total cholesterol level was found to be 141.09 ± 48.59 mg/dL, which is considerably lower than the generally accepted desirable limit of <200 mg/dL. While at first glance this might appear favorable, it is essential to consider that acute illness is often associated with a transient suppression of cholesterol levels due to the systemic inflammatory response, known as the “lipid paradox.” In such scenarios, cholesterol levels measured during hospitalization may not accurately reflect baseline lipid status, and thus, their interpretation requires caution. The lipid profile might not reflect the patients’ chronic atherogenic status and may underestimate long-term cardiovascular risk [14]. Therefore, follow-up lipid evaluation after recovery, along with consideration of lifestyle, medication history, and dietary habits, is essential for guiding secondary prevention strategies. Moreover, intensive management with statins during the episode might affect these findings.

Moreover, the results raise an important clinical point-stroke can and often does occur even in the absence of traditional dyslipidemia. This underscores the need for a broader focus on vascular risk assessment, including emerging biomarkers and non-lipid risk factors, to more comprehensively evaluate and manage stroke risk in diverse patient populations.

In this study, we found notable alterations in circadian rhythm markers among AIS patients. Elevated levels of salivary cortisol were noted in the cases. This result failed to reach statistical significance, likely due to high interpatient variability. In contrast, urinary melatonin concentrations were significantly lower in stroke patients (7.18 ± 1.91 ng/mL) than in controls (14.26 ± 1.98 ng/mL), and this difference was highly significant (p < 0.0001), suggesting impaired pineal function and circadian disruption.

These findings are in line with evidence from the TABASCO prospective cohort study, in which Tene et al. found that elevated bedtime salivary cortisol levels following stroke were associated with greater brain atrophy and long-term cognitive decline. The study showed that individuals with persistently high cortisol levels had significantly worse memory and executive functioning scores over 24 months. The relationship between cortisol and cognitive performance was particularly pronounced in carriers of the ApoE4 allele, suggesting a gene-environment interaction that may amplify cortisol’s neurodegenerative effects [15].

While the TABASCO study focused primarily on cortisol, our study complements those findings by showing that reduced melatonin levels also characterize the post-stroke hormonal milieu. Melatonin plays a critical role in sleep regulation and neuroprotection through antioxidant, anti-inflammatory, and antiapoptotic pathways. A significant reduction in melatonin among stroke patients may, therefore, contribute to impaired circadian entrainment, poor sleep quality, and suboptimal neurological recovery [16].

Taken together, these results suggest that dysregulation of circadian hormones-namely, elevated cortisol and suppressed melatonin-is a key pathophysiological feature in stroke patients. These hormonal shifts may not only serve as biomarkers of injury severity but also represent potential therapeutic targets that should be studied in future trials. Interventions aimed at restoring circadian integrity, such as melatonin supplementation, light therapy, or stress modulation strategies, could enhance recovery trajectories and long-term outcomes in post-stroke populations.

ABPM in our study revealed significant alterations in diurnal BP variation among stroke patients compared to controls. The stroke cases demonstrated a significantly higher number of non-dipper and reverse dipper patterns as compared to the control group (χ² = 37.81, p < 0.0001). This pronounced shift toward non-dipping and reverse dipping patterns in stroke patients points to impaired autonomic regulation and increased vascular rigidity-features common in individuals with longstanding hypertension, diabetes, and subclinical atherosclerosis. The presence of a reverse dipping profile, though rare, is particularly concerning, as it is associated with increased risk of silent cerebral infarctions, white matter lesions, and recurrent strokes.

Our findings are consistent with those of Kario et al., who reported in the Japan Ambulatory Blood Pressure Monitoring Prospective (JAMP) study that nighttime BP patterns are strong predictors of stroke and cardiovascular events [17]. They highlighted that individuals with non-dipping or reverse dipping phenotypes had significantly higher rates of cerebrovascular events, independent of daytime BP levels. This underscores the prognostic superiority of 24-hour monitoring over clinic-based measurements.

In the context of stroke recovery, disrupted circadian BP patterns may also influence perfusion dynamics and neurological outcomes. Though our subgroup analyses did not yield statistically significant associations between ABPM categories and MRS scores at discharge, these might be limited due to the small sample size.

The integration of ABPM into stroke assessment protocols offers valuable insights not only into BP control but also into vascular health, autonomic function, and chronobiological stability. Identifying non-dippers and reverse dippers early could inform individualized therapeutic strategies, including nocturnal antihypertensive therapy, salt restriction, or chronotherapy.

Association of ABPM patterns with stroke outcomes (MRS, GCS, and NIHSS)

We explored the relationship between ABPM patterns-dipper, non-dipper, and reverse dipper-and key stroke outcomes. While the associations were not statistically significant, notable trends emerged that align with prior evidence on the prognostic implications of altered circadian BP regulation.

In our study, stroke patients with a dipper BP pattern had a slightly higher likelihood of achieving better functional outcomes at discharge, as reflected in MRS scores. Specifically, 30.7% of dippers achieved MRS scores of 1 or 2, compared to only 16.2% among non-dippers. Interestingly, all three patients with a reverse dipping pattern had poor outcomes (MRS 3-5). However, due to the small number of reverse dippers (n = 3), statistical comparison was limited (χ² = 3.443; p = 0.9036).

NIHSS-based stroke severity showed a more mixed distribution across ABPM patterns. Minor stroke was more prevalent in dippers (43.6%) than in non-dippers (32.4%) and was absent in reverse dippers. Conversely, moderate-to-severe strokes were more common in the reverse dipping subgroup. However, statistical analysis revealed no significant association between ABPM pattern and NIHSS severity category (χ² = 2.411; p = 0.6607).

These findings are conceptually aligned with data from Ding et al., who reported that reverse dipping was associated with worse one-year functional outcomes in patients with ischemic stroke or transient ischemic attack. However, this significance disappeared after adjusting for confounders in their study. Although our results did not reach statistical significance, likely due to sample size limitations, the observed trends reinforce the hypothesis that disrupted circadian BP patterns are linked with less favorable neurological and functional recovery [18].

In unison, these findings support the hypothesis that markers of circadian rhythm-whether hormonal or hemodynamic-are intimately linked with the prognosis and recovery trajectory of ischemic stroke. The disruption of these rhythms may serve as both a consequence of cerebral insult and a contributing factor to poor outcomes, creating a self-reinforcing pathophysiological cycle.

Given these insights, this study not only strengthens the role of cortisol, melatonin, and ABPM profiles as potential prognostic markers but also paves the way for future exploration of circadian-targeted therapeutic strategies. Interventions aimed at restoring hormonal and hemodynamic rhythms-such as melatonin supplementation, sleep optimization, light therapy, or timed antihypertensive administration-may enhance recovery and functional independence in stroke survivors.

Limitations of the study

Despite yielding clinically relevant findings, this study had several limitations that must be acknowledged. Although the calculated sample size of 150 participants (75 cases and 75 controls) provided a reasonable basis for analysis [19], it may have been underpowered to detect subtle or complex interactions, particularly in subgroup analyses such as reverse dipper ABPM patterns or cortisol stratification across outcome scores.

Controls were recruited by convenience sampling and were not matched for vascular risk factors. Multivariate analysis was not performed to adjust for hypertension or dipper status, which may confound associations.

Results from a single-center study limit the generalizability to broader populations and different ethnic and geographic groups. Furthermore, salivary cortisol and urinary melatonin were assessed at a single time point, which may not capture circadian fluctuations accurately; future studies should implement serial sampling or circadian profiling to overcome this limitation. Confounders such as sleep quality; clinical frailty; stress; medications such as corticosteroids, beta-blockers, and sleep aids, which can affect the levels of these biomarkers; and light exposure may have influenced hormonal levels and were not fully controlled [20]. And lastly, the lack of long-term follow-up restricts insights into the predictive value of circadian markers and ABPM patterns for outcomes like recovery, recurrence, or cognitive decline.

## Conclusions

This study investigated the relationship between circadian rhythm markers (urinary melatonin and salivary cortisol) and ABPM in AIS outcomes at a northern Indian tertiary care center. The findings revealed significantly reduced urinary melatonin levels in stroke patients compared to controls, showing strong negative correlations with stroke severity (NIHSS) and functional disability (MRS). While salivary cortisol levels were elevated in stroke patients, they showed variable patterns across outcome categories, suggesting a complex role of the hypothalamic-pituitary-adrenal axis. ABPM demonstrated that most stroke patients deviated from normal "dipper" patterns, showing "non-dipper" and "reverse dipper" profiles, though these variations did not reach statistical significance in relation to clinical outcomes. The study concluded that circadian markers could serve as valuable prognostic indicators in acute stroke care, warranting further large-scale studies to validate their clinical utility in personalized treatment planning.

## References

[1] Chitimus DM, Popescu MR, Voiculescu SE, Panaitescu AM, Pavel B, Zagrean L, Zagrean AM (2020). Melatonin's impact on antioxidative and anti-inflammatory reprogramming in homeostasis and disease. Biomolecules.

[2] Andrabi SS, Parvez S, Tabassum H (2015). Melatonin and ischemic stroke: mechanistic roles and action. Adv Pharmacol Sci.

[3] Alghamdi BS (2018). The neuroprotective role of melatonin in neurological disorders. J Neurosci Res.

[4] Lin HW, Lee EJ (2009). Effects of melatonin in experimental stroke models in acute, sub-acute, and chronic stages. Neuropsychiatr Dis Treat.

[5] Grossman E, Laudon M, Zisapel N (2011). Effect of melatonin on nocturnal blood pressure: meta-analysis of randomized controlled trials. Vasc Health Risk Manag.

[6] Jonas M, Garfinkel D, Zisapel N, Laudon M, Grossman E (2003). Impaired nocturnal melatonin secretion in non-dipper hypertensive patients. Blood Press.

[7] Barugh AJ, Gray P, Shenkin SD, MacLullich AM, Mead GE (2014). Cortisol levels and the severity and outcomes of acute stroke: a systematic review. J Neurol.

[8] Wang JJ, Xu FB, Hu S, Xu YM, Wang XZ (2024). Identification and validation of cortisol-related hub biomarkers and the related pathogenesis of biomarkers in ischemic stroke. Brain Behav.

[9] Akhtar N, Al-Jerdi S, Kamran S (2021). Night-time non-dipping blood pressure and heart rate: an association with the risk of silent small vessel disease in patients presenting with acute ischemic stroke. Front Neurol.

[10] Kakaletsis N, Ntaios G, Milionis H (2015). Prognostic value of 24-h ABPM in acute ischemic stroke for short-, medium-, and long-term outcome: a systematic review and meta-analysis. Int J Stroke.

[11] Maciejczyk M, Bielas M, Zalewska A, Gerreth K (2021). Salivary biomarkers of oxidative stress and inflammation in stroke patients: from basic research to clinical practice. Oxid Med Cell Longev.

[12] Umemura T, Kawamura T (2014). Effect of diabetes on stroke symptoms and mortality: lessons from a recent large population-based cohort study. J Diabetes Investig.

[13] Rosenson RS (1993). Myocardial injury: the acute phase response and lipoprotein metabolism. J Am Coll Cardiol.

[14] Zhou X, Yang Q (2020). From hemorrhagic stroke to lipid paradox: a double-hit hypothesis underlying low low-density lipoprotein cholesterol related cardiovascular risk—a narrative review. J Bio-X Res.

[15] Tene O, Hallevi H, Korczyn AD (2018). The price of stress: high bedtime salivary cortisol levels are associated with brain atrophy and cognitive decline in stroke survivors. Results from the TABASCO prospective cohort study. J Alzheimers Dis.

[16] Zisapel N (2018). New perspectives on the role of melatonin in human sleep, circadian rhythms and their regulation. Br J Pharmacol.

[17] Kario K, Hoshide S, Mizuno H (2020). Nighttime blood pressure phenotype and cardiovascular prognosis: practitioner-based nationwide JAMP study. Circulation.

[18] Ding X, Zhou Y, Pan Y (2023). Dipping pattern and 1-year stroke functional outcome in ischemic stroke or transient ischemic attack. Clin Exp Hypertens.

[19] Kamal K, Tewari J, Bharti V (2023). Serum vitamin D level as a risk factor and prognostic marker for acute ischemic stroke: a case-control study at a tertiary care centre in Northern India. Cureus.

[20] Pedregosa BC II, Liban MD, Batino LK, Escabillas CG, Villaraza SG, Navarro JC, Qureshi AI (2025). Premorbid clinical frailty and outcomes after intravenous thrombolysis in patients with acute ischemic stroke—a multicenter retrospective cohort study. J Stroke Med.

